# Supplementary material for: ICM conversion to epiblast by FGF/ERK inhibition is limited in time and requires transcription and protein degradation
Source: Sci Rep. 2017 Sep 25;7:12285. doi: 10.1038/s41598-017-12120-0 (PMC5612930; doi:10.1038/s41598-017-12120-0)
Supplement: Supplementary file 1 — Supplementary information [file 41598_2017_12120_MOESM1_ESM.pdf]

Supplementary information

ICM conversion to epiblast by FGF/ERK inhibition is limited in time and requires transcription and protein degradation

Sylvain Bessonnard[1], Sabrina Coqueran[1], Sandrine Vandormael-Pournin[1], Alexandre Dufour[32], Jérôme Artus[1,3, 4, 5] and Michel Cohen-Tannoudji[1, 5]

[1] :Institut Pasteur, CNRS, Unité de Génétique Fonctionnelle de la Souris, UMR 3738, Department of Developmental & Stem Cell Biology, 25 rue du docteur Roux, F-75015 Paris Cedex.

[2] : Institut Pasteur, Bioimage Analysis Unit, CNRS UMR 3691, Paris, France.

[3] : present address, INSERM UMR935, Paul Brousse Hospital, University Paris Sud , Villejuif, France

[4] : present address, Faculty of Medicine, Kremlin-Bicêtre, University Paris Sud, Paris Saclay, France

[5] : Co-author

Running title: Temporal dynamics of ICM specification

Corresponding authors: Jérôme Artus, INSERM U935, 7 rue Guy Mocquet, 94802 Villejuif Cedex; France; E-mail: jerome.artus@u-psud.fr; Phone: +33 1 49 58 33 35. Michel Cohen-Tannoudji, Unité de Génétique Fonctionnelle de la Souris, Department of Developmental & Stem Cell Biology, 25 rue du docteur Roux, F-75015 Paris, France; E-mail: m-cohen@pasteur.fr; Phone: 33 1 45 68 84 86; Fax: 33 1 45 68 86 34; web site: https://research.pasteur.fr/en/team/group-michel-cohen-tannoudji/.

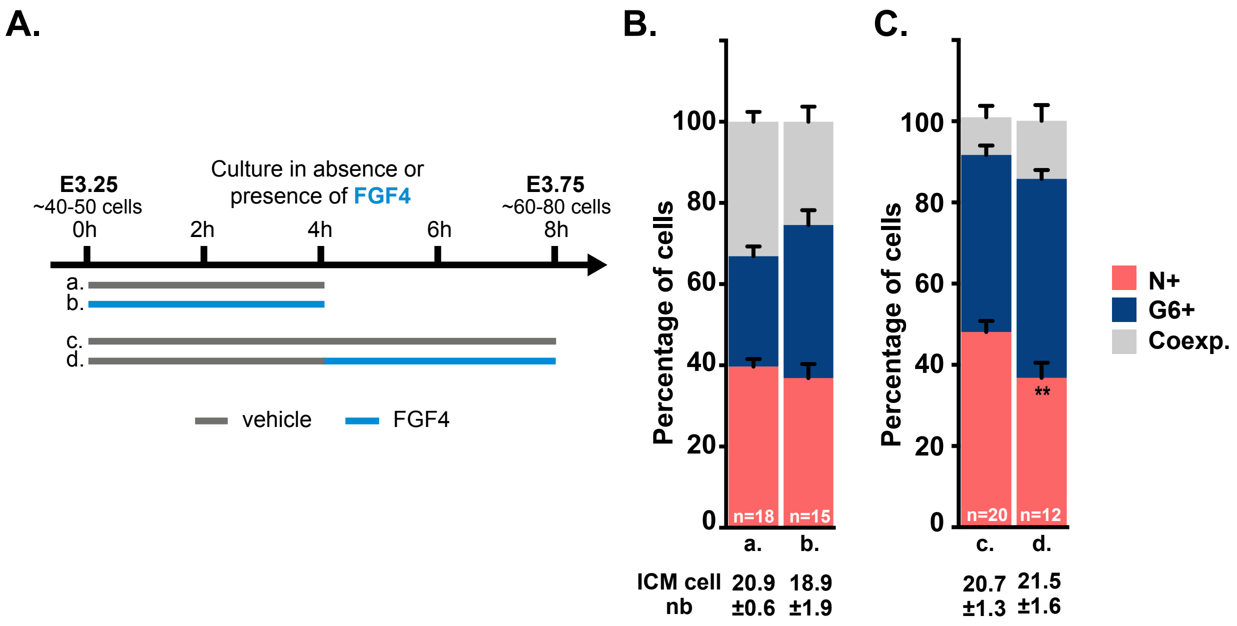

Supplementary Figure S1 (related to Figures 3 and 4). Effect on ICM specification of exogenous FGF 4 stimulation between E3.25 and E3.75. (A) Schematic of the time schedule of vehicle (grey) and FGF4 (blue) treatment. **(B-C)** Distribution of ICM cells expressing NANOG (N+, red), GATA6 (G6+, blue) or both markers (Coexp., grey) in embryos cultured in presence or absence of FGF4. Control embryos plotted in Figure S2B are similar to Figure 1B. Error bars indicate SEM. *n*, number of embryos analyzed. Statistical Mann–Whitney tests are indicated when significant (**, $p < 0.01$).

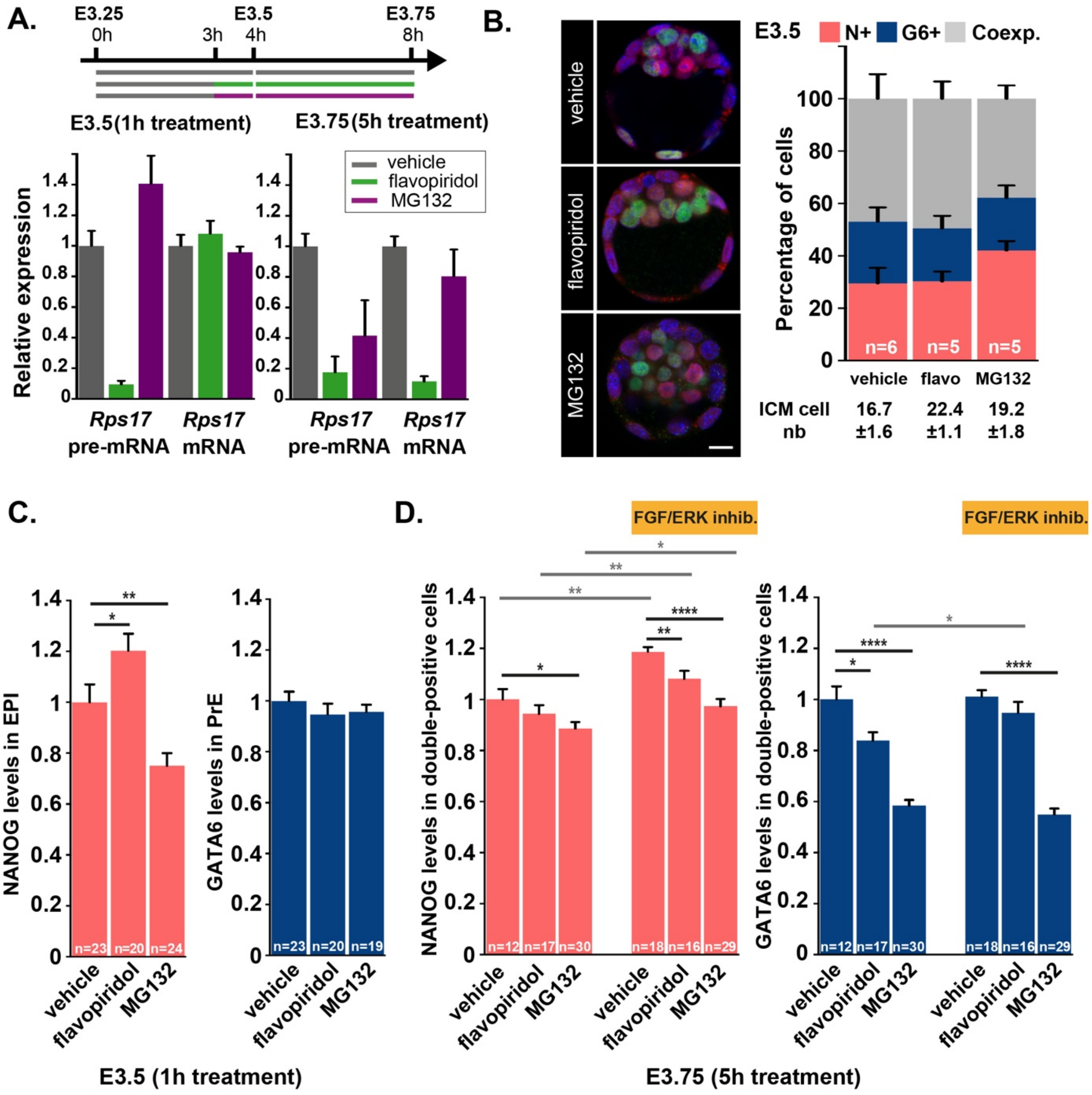

Supplemental Figure S2 (related to Figure 5). Effect of modulating transcription/proteasome activity during ICM specificationw. (A) Schematic of the time schedule of inhibitor treatment. Green, purple and grey lines indicate the culture periods in the presence of flavopyridol, MG132 and DMSO (vehicle), respectively. RT-qPCR expression analysis of *Rps17* pre-mRNA and *Rps17* mRNA in embryos cultured 1h and 5h with/without the drugs. **(B)** Immunodetection of NANOG (green) and GATA6 (red) in embryos cultured 1h (from 3h to 4h) in the presence/absence of drugs. Pictures correspond to a projection of 5 confocal optical slices. Scale bar: 20µm. Distribution of ICM cells expressing NANOG (N+, red), GATA6 (G6+, blue) or both markers (Coexp., grey) in embryos cultured for the indicated period of times. Error bars indicate SEM. *n*, number of embryos analyzed. **(C)** Quantification of NANOG levels

in Epi (NANOG-positive) progenitors and of GATA6 levels inPrE (GATA6-positive) in E3.5 embryos after 1 hours of treatment. **(D)** Quantification of NANOG and GATA6 levels in co-expressing ICM cells in E3.75 embryos after 5 hours of treatment. Error bars indicate SEM. *n*, number of cells analyzed. Statistical Mann–Whitney tests are indicated when significant (*, $p < 0.05$; **, $p < 0.01$;, ****, $p<0.0001$).
